# Development and clinical validation of a dual ddPCR assay for detecting carbapenem-resistant *Acinetobacter baumannii* in bloodstream infections

**DOI:** 10.3389/fmicb.2024.1338395

**Published:** 2024-03-05

**Authors:** Xiaoxia Kou, Detu Zhu, Yandong Zhang, Liyan Huang, Jiawei Liang, Ziman Wu, Ze Liu, Chushi Guan, Lin Yu

**Affiliations:** ^1^Department of Laboratory, The Key Laboratory of Advanced Interdisciplinary Studies Center, The First Affiliated Hospital of Guangzhou Medical University, Guangzhou, Guangdong, China; ^2^Guangzhou Key Laboratory for Clinical Rapid Diagnosis and Early Warning of Infectious Diseases, KingMed School of Laboratory Medicine, Guangzhou Medical University, Guangzhou, China; ^3^Biologics Test and Evaluation Center, Guangzhou Laboratory, Guangzhou, China

**Keywords:** ddPCR, *Acinetobacter baumannii*, carbapenemase, bloodstream infection, antibiotic resistance

## Abstract

**Objective:**

*Acinetobacter baumannii* (*A. baumannii*, AB) represents a major species of Gram-negative bacteria involved in bloodstream infections (BSIs) and shows a high capability of developing antibiotic resistance. Especially, carbapenem-resistant *Acinetobacter baumannii* (CRAB) becomes more and more prevalent in BSIs. Hence, a rapid and sensitive CRAB detection method is of urgent need to reduce the morbidity and mortality due to CRAB-associated BSIs.

**Methods:**

A dual droplet digital PCR (ddPCR) reaction system was designed for detecting the antibiotic resistance gene OXA-23 and AB-specific gene *gltA*. Then, the specificity of the primers and probes, limit of detection (LOD), linear range, and accuracy of the assay were evaluated. Furthermore, the established assay approach was validated on 37 clinical isolates and compared with blood culture and drug sensitivity tests.

**Results:**

The dual ddPCR method established in this study demonstrated strong primer and probe specificity, distinguishing CRAB among 21 common clinical pathogens. The method showed excellent precision (3 × 10^−4^ ng/μL, CV < 25%) and linearity (OXA-23: *y* = 1.4558*x* + 4.0981, *R*^2^ = 0.9976; *gltA*: *y* = 1.2716*x* + 3.6092, *R*^2^ = 0.9949). While the dual qPCR LOD is 3 × 10^−3^ ng/μL, the dual ddPCR’s LOD stands at 3 × 10^−4^ ng/μL, indicating a higher sensitivity in the latter. When applied to detect 35 patients with BSIs of AB, the results were consistent with clinical blood culture identification and drug sensitivity tests.

**Conclusion:**

The dual ddPCR detection method for OXA-23 and *gltA* developed in this study exhibits good specificity, excellent linearity, and a higher LOD than qPCR. It demonstrates reproducibility even for minute samples, making it suitable for rapid diagnosis and precision treatment of CRAB in BSIs.

## 1 Introduction

Bloodstream infection (BSI) is one of the main causes of lethality around the world (Viscoli, 2016; Liu et al., 2023). Approximately 95% of the causative agents for BSIs are bacteria (Smith et al., 2021; Yu et al., 2021; Fakih et al., 2022; Noster et al., 2022). Even worse, the drug resistance of bacteria exacerbates the mortality rate. Therefore, a rapid detection method of pathogens and their resistance is urgently required to reduce the morbidity and mortality associated with BSIs (Garnacho-Montero et al., 2015; Dao et al., 2020; Paramita et al., 2020; Yang et al., 2021). *Acinetobacter baumannii* (*A. baumannii*, AB) is one of the major microorganisms that cause BSIs and a significant cause of hospital-acquired infections (Bartual et al., 2005; Nasr, 2020).

Recently, with the widespread use of broad-spectrum antibiotics, the prevalence of carbapenem-resistant AB (CRAB) has increased, challenging the current clinical treatments and resulting in higher mortality rates (Nasr, 2020). According to CHINET statistics, the percentages of AB resistant to imipenem and meropenem in China have risen from 31.7 and 39.9% in 2006 to 71.5 and 72.3% in 2021, respectively (Hu et al., 2018). Abroad, the prevalence of MDRAB in Iran increased from 50% in 2001–2007 to 74% in 2010–2015 (Rezai et al., 2023). Previously, we identified blaOXA-23 as the dominant carbapenemase gene type in CRAB isolated from clinical patients with BSIs at our hospital from 2018 to 2022. Globally, the blaOXA23 gene has a high correlation with the carbapenem-resistant phenotype of AB, making it a powerful predictor for carbapenem resistance (Piperaki et al., 2019; Zhang et al., 2021; Abouelfetouh et al., 2022). These findings are consistent with prior research studies conducted by our team and the others (Oliveira et al., 2019; Douraghi et al., 2020; Koirala et al., 2020; Cui et al., 2021; Lombes et al., 2022; Zhang et al., 2022).

Droplet digital PCR (ddPCR) is one of the emerging nucleic acid detection and quantitation technologies. It separates the template DNA into tens of thousands of independent reaction units for amplification, so it can detect the target gene without being interfered by the complex environment in the blood (Váňová et al., 2021; Zheng et al., 2021; Del Arco et al., 2022; Gao et al., 2022). Unlike real-time quantitative PCR (qPCR), ddPCR does not rely on the amplification curve cycle thresholds (Ct values) and standard curves. It can achieve absolute quantitative analysis and has better sensitivity and reproducibility (Liu et al., 2019; Kojabad et al., 2021).

Therefore, this study proposes to establish a method for dual detecting the AB-specific gene *gltA* and the carbapenem resistance gene OXA-23 through ddPCR, thus providing a useful tool for rapid diagnosis and precise medication of AB in BSIs.

## 2 Materials and methods

### 2.1 Sample source

From January 2018 to December 2022, 35 strains of AB were isolated from clinical blood cultures at the First Affiliated Hospital of Guangzhou Medical University. Repetitive strains from the same patient were excluded. These strains are comprised of seven carbapenem-sensitive *Acinetobacter baumannii* (CSAB), 12 multidrug-resistant *Acinetobacter baumannii* (MDRAB), and 16 extensively drug-resistant *Acinetobacter baumannii* (XDRAB). Relevant clinical information was recorded for each strain. We also included 21 common types of fungal, bacterial, and viral strains. Sample isolation and culture were performed following the National Clinical Laboratory Procedure (Fourth Edition).

### 2.2 Experimental methods

#### 2.2.1 Bacterial DNA extraction

Bacterial DNA was extracted using a column-based method. The concentration and purity of the extracted DNA were assessed and recorded using the NanoDrop2000 spectrophotometer, and the DNA was stored at −20°C for subsequent uses.

#### 2.2.2 Duplex ddPCR reaction system

A dual ddPCR assay was performed using the QX200 TM Droplet Digital PCR System (Bio-rad, United States) to detect AB and its carbapenemase gene simultaneously in one chip following the manufacturer’s protocol. This included 10 μL of ddPCR TM Supermix for probes (No dUTP), 1 μL of each of the forward and reverse primers for OXA-23/*gltA* (10 μM), 0.5 μL of the OXA-23/*gltA* probe (10 μM), and 2 μL of target DNA, with the volume made up to 20 μL with distilled water. The amplification program was: 95°C for 10 min for pre-denaturation; 94°C for 30 s for denaturation, 60°C for 1 min for annealing, for 40 cycles; 98°C for 10 min for enzyme deactivation, and stored at 4°C.

#### 2.2.3 Design of primers and probes

Primers and TaqMan probes were designed using Primer Premier and synthesized by Shanghai Bioengineering Co., Ltd. Based on the gene sequences of OXA-23 and *gltA*, three different sets of primers were designed. The 5′ end of the probe designed on the OXA-23 gene was labeled with the 6-carboxy-fluorescein (FAM), and the 5′ end of the probe designed on the *gltA* gene was labeled with the hexachloro fluorescein (HEX). A non-fluorescent quencher was added to the 3′ end of the probe. The sensitivity and specificity were evaluated by qPCR and ddPCR, respectively.

#### 2.2.4 Optimization of dual ddPCR reaction system

The amplification efficiency and fluorescence intensity of the ddPCR reaction system is related to the concentration of primer probes, the ratio between the two primers, and the annealing temperature, among other factors. Therefore, this experiment optimized primer concentration, primer concentration ratio, and annealing temperature.

#### 2.2.5 Linear range test and LOD judgment

To evaluate the limit of detection (LOD) and linear range of the established reaction system, we selected a strain of carbapenem-resistant *Acinetobacter baumannii* (CRAB). Upon determining its initial concentration (300 ng/μL), we performed a 10-fold serial dilution, creating a concentration gradient ranging from 3.0 × 10^2^ to 3.0 × 10^−4^ ng/μL. Distilled water was utilized as a negative control. Each concentration gradient was repeated three times. Following this, the sensitivities of dual qPCR and dual ddPCR methods were comparatively analyzed.

#### 2.2.6 Accuracy test

To evaluate the reproducibility of the dual ddPCR, repeated experiments were performed with CRAB bacterial fluid at four different concentrations (3 × 10^−1^, 3 × 10^−2^, 3 × 10^−3^, and 3 × 10^−4^ ng/μL). Each concentration was repeated three times. The CV was calculated based on the number of positive droplets obtained from the amplification of each target. The reproducibility and stability of the method are evaluated by the standard of CV < 25% for each concentration [European Network of GMO Laboratories (ENGL), 2015].

#### 2.2.7 Clinical specimen verification

In this study, we selected 37 venous blood samples from patients who tested positive and negative for AB in clinical blood cultures for examination. The DNA from these venous blood samples, which included 1 negative sample, 1 *Escherichia coli*, 7 CSAB, and 28 CRAB, was extracted using a DNA extraction kit. This extracted DNA was then amplified using the established duplex ddPCR method. Results were compared with blood culture and drug sensitivity test.

## 3 Results

### 3.1 Primer set screening

**Figure 1:**
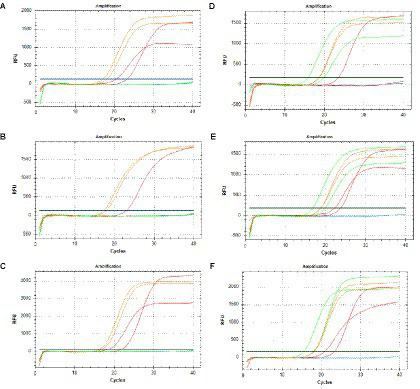
Screening of primers and probes with qPCR: OXA-23 **(A–C)** and *gltA* **(D–F)** (Green, orange, and red amplification curves were represented CSAB, MDRAB, and XDRAB, respectively. Blue amplification curves were represented blank control).

Three sets of primers and probes synthesized for targeting OXA-23 and *gltA* were screened. The first primer set failed to amplify the *gltA* gene of one strain of CSAB (Figures 1A,D). The second primer set failed to amplify the OXA-23 resistance gene of one XDRAB strain (Figures 1B,E). The third primer set successfully amplied the *gltA* gene of all AB strains, and the OXA-23 resistance gene of all CRAB strains (Figures 1C,F). Additionally, for the same sample, the Ct value of the amplification by the third primer set was smaller than those of the above two primer sets (Supplementary Tables 1, 2), indicating higher sensitivity. Therefore, the third primer set was chosen for the reaction system (Supplementary Table 3).

### 3.2 Specificity evaluation of primers and probes

**Figure 2:**
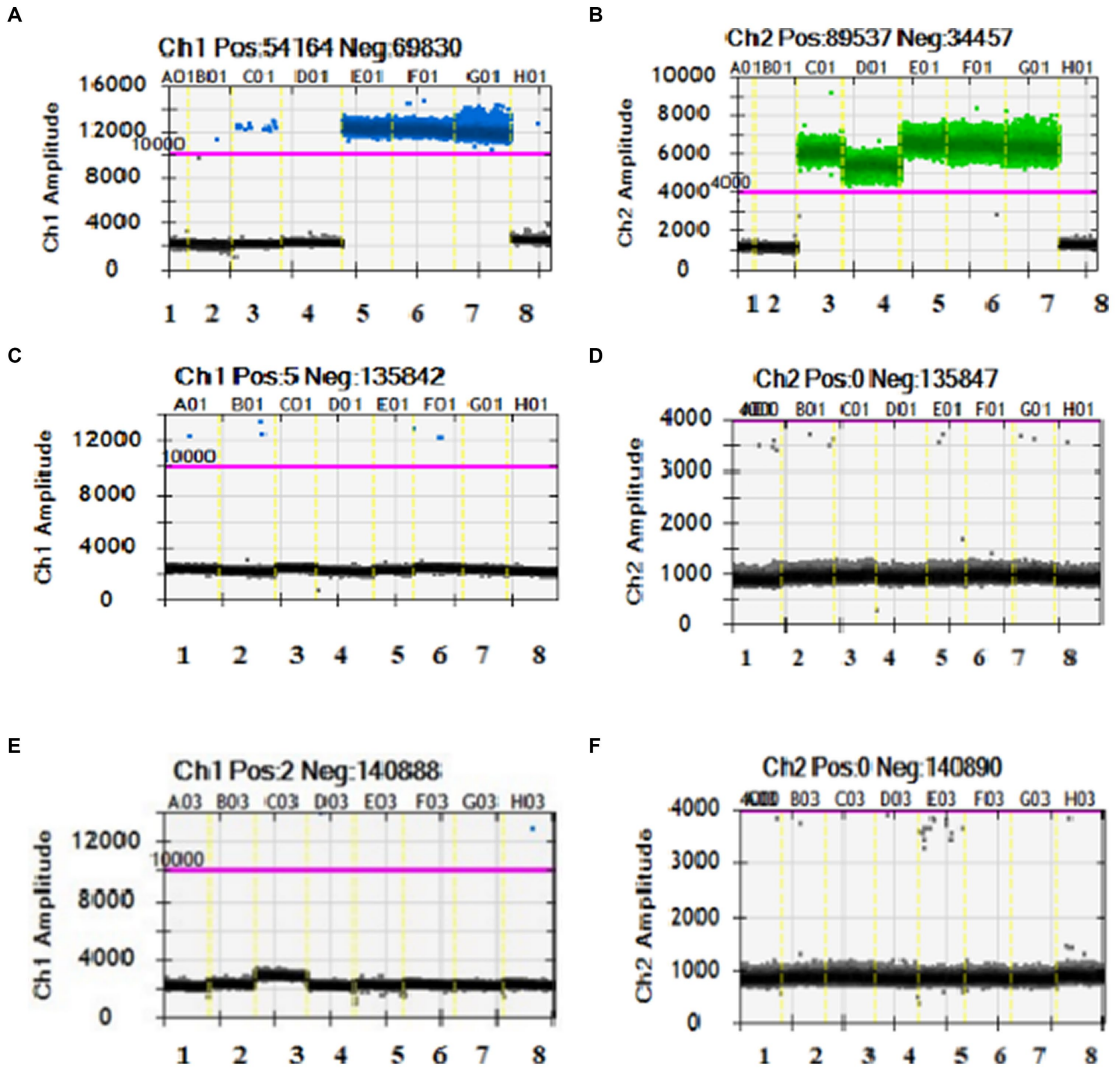
Specificity results of the duplex PCR (More than two droplets of blue drops or green drops above purple threshold line indicate carrying *blaOXA-23* or *gltA gene,* respectively. If the number of droplets is less than or equal to 2, it will be judged as a negative result.). Panels **(A,B)** (both from one to eight) are H_2_O, *Escherichia coli*, CRAB, CSAB, MDRAB-1, MDRAB-2, XDRAB-1, *Staphylococcus aureus*. CSAB has only green signals. CRAB, MDRAB-1, MDRAB-2 and XDRAB-1 have both blue and green signals. Panels **(C,D)** (both from one to eight) are *Corynebacterium striatum*, *Pseudomonas aeruginosa*, *Enterococcus faecium*, *Enterococcus faecalis*, *Staphylococcus warneri*, *Staphylococcus capitis*, *Streptococcus pneumoniae*, *Serratia marcescens*. They all have negative results. Panels **(E,F)** (both from one to eight) are *Arestreptococcus pyogenes*, *Klebsiella pneumoniae*, *Moraxella catarrhalis*, *Adenovirus*, *Candida albicans*, *Candida tropicalis*, HBV, CMV. They all have negative results.

Using the established qPCR and ddPCR reaction systems, we carried out specificity tests for the third primer set on 21 common clinical samples of fungi, bacteria, and viruses. As expected, there is no amplification reaction for these samples except for the positive control CRAB DNA, demonstrating good specificity (Figure 2; Supplementary Tables 4 5).

### 3.3 Optimization of the dual ddPCR reaction system

#### 3.3.1 Optimization of annealing temperature in dual ddPCR

**Figure 3:**
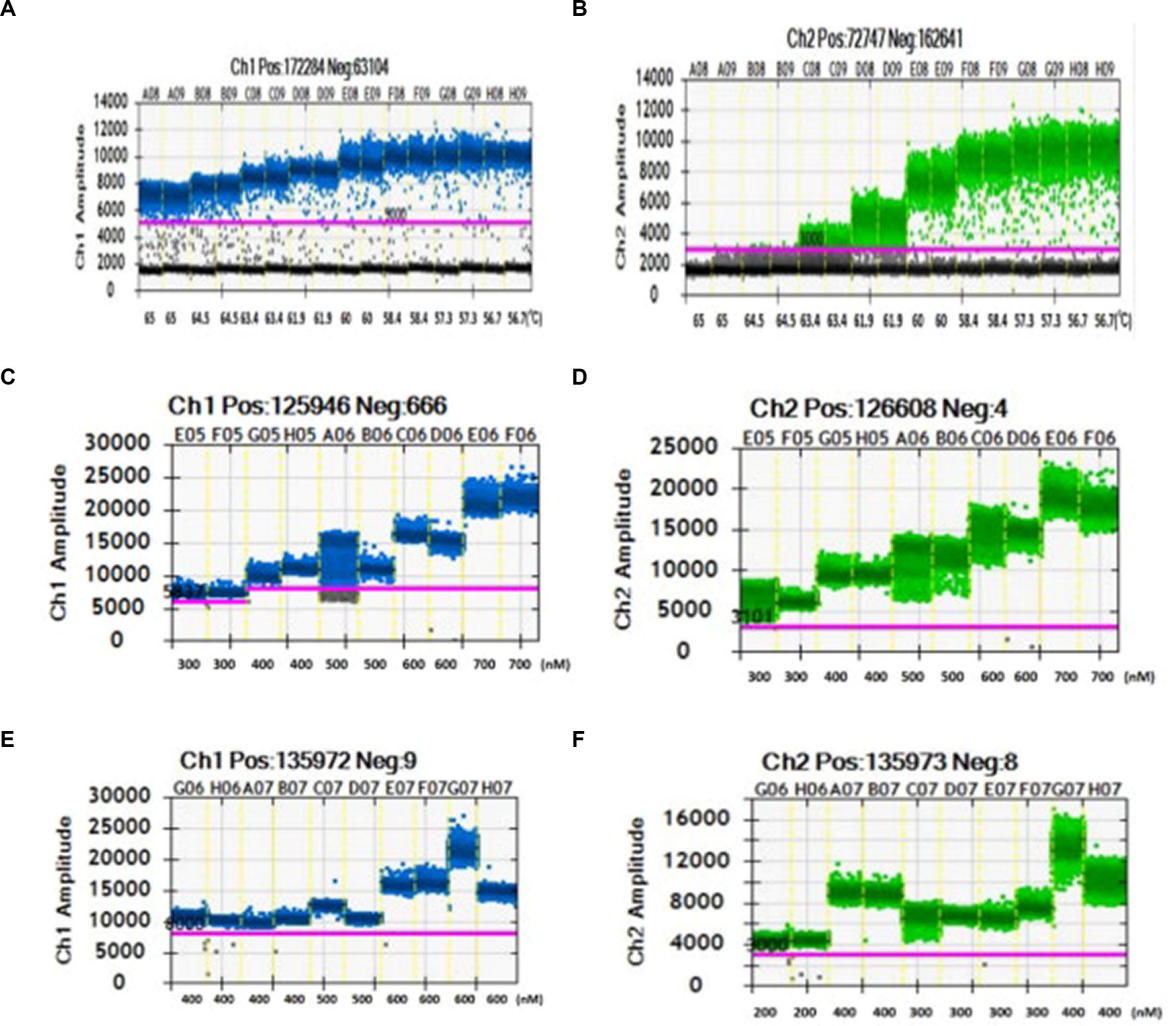
Optimization of the Dual ddPCR System (Blue for OXA-23; Green for *gltA*): **(A,B)** Temperature optimization results (the temperature in the two images from left to right were 65, 64.5, 63.4, 61.9, 60, 58.4, 57.3, and 56.7°C). **(C,D)** Primers concentration optimization results (the primer concentrations in the two images from left to right were 300, 400, 500, 600, and 700 nM). **(E,F)** Primer ratio optimization results (the primer ratio in the two images from left to right were 400:200 nM, 400:400 nM, 500:300 nM, 600:300 nM, and 600:400 nM. The primer concentrations in the panel **(A)** from left to right were 400, 400, 500, 600, and 600 nM. The primer concentrations in the panel **(B)** from left to right were 200, 400, 300, 300, and 400 nM).

The results of the dual ddPCR annealing temperature optimization experiment are shown in Figures 3A,B. The variation in annealing temperature had a noticeable impact on *gltA*. When the annealing temperature was set to 64.5 or 65°C, the dual ddPCR reaction system did not amplify *gltA*. At annealing temperatures of 61.9 and 63.4°C, the positive and negative droplets could not be clearly distinguished. However, when the annealing temperature was between 56.7 and 60°C, the positive droplets could be clearly distinguished from the negative droplets. Therefore, 56.7°C was ultimately chosen as the optimal annealing temperature.

#### 3.3.2 Optimization of dual ddPCR primer concentration

With the primer concentration ratio of OXA-23 and *gltA* fixed at 1:1 in the dual ddPCR system, the two pairs of primers underwent concentration optimization. The results are shown in Figures 3C,D. Comparing the amplification results in the figure, it can be seen that the amplification effect is optimal at a concentration of 400 nM. Moreover, positive droplets are more concentrated compared to other primer concentrations. Therefore, 400 nM was chosen as the optimal primer concentration in the reaction system.

#### 3.3.3 Optimization of dual ddPCR OXA-23: *gltA* primer concentration ratio

The results of the optimization of the dual ddPCR primer concentration ratio is shown in Figures 3E,F. The distribution of positive and negative droplet numbers generated after amplification in systems with different primer concentration ratios differs. When the primer concentration ratios were 500:300 and 600:400 nM, the cluster of positive droplets was scattered, hard to be distinguished, and lacked reproducibility. Meanwhile, when the primer concentration ratios were 400:200, 400:400, and 600:300 nM, the cluster of positive droplets was concentrated. Furthermore, the reaction system with a primer concentration ratio of 400:400 nM had a higher fluorescence intensity of positive droplets, which could be clearly distinguished from negative droplets.

### 3.4 Linear range test and LOD judgment

The dual qPCR and ddPCR amplification systems were separately used to detect OXA-23 and *gltA* in CRAB with varying gradient concentrations. The LOD of ddPCR is higher than that of qPCR (Table 1).

**Table 1:** LOD results of OXA-23 and *gltA* between ddPCR and qPCR.

Theoretical concentration (ng/μL)		0.0003	0.003	0.03	0.3	3	30	300
OXA-23	qPCR (Ct value)	N.D.	36.10	32.05	24.36	19.68	16.10	13.16
	ddPCR (copies/μL)	0.08	3.6	65.7	2,165	11,300	>1,000,000	>1,000,000
gltA	qPCR (Ct value)	N.D.	37.34	33.20	25.64	21.12	17.58	14.52
	ddPCR (copies/μL)	0.17	2	37.1	1,113	11,300	>1,000,000	>1,000,000

N.D. means no amplification.

**Figure 4:**
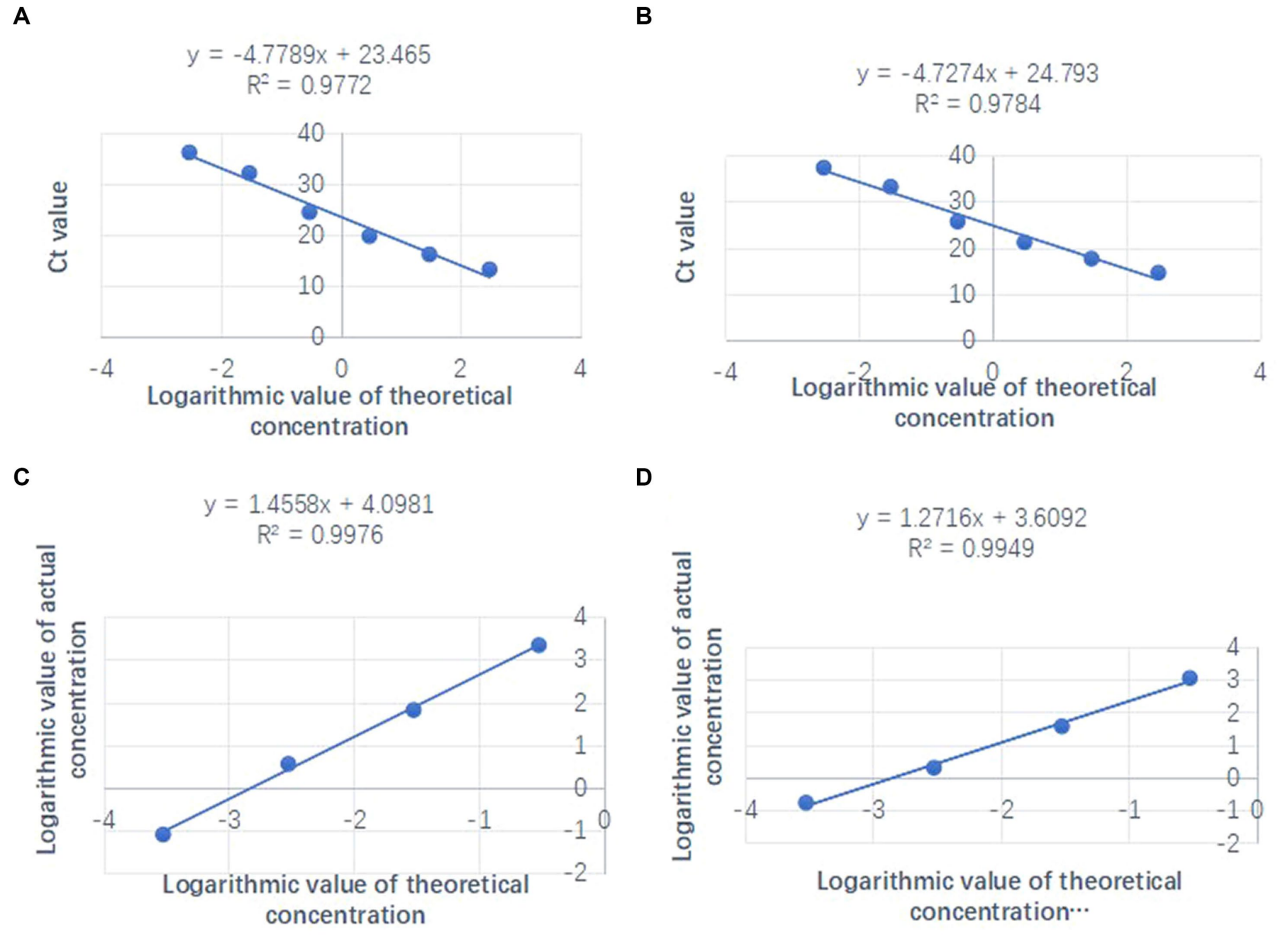
The linearity of qPCR and ddPCR: **(A)** OXA-23-duplex qPCR standard curve. **(B)** *gltA*-duplex qPCR standard curve (the target gene concentration corresponding to the amplification curve is 3 from left to right were 300, 30, 3, 3 × 10^−1^, 3 × 10^−2^, and 3 × 10^−3^ ng/μL). **(C)** OXA-23-duplex ddPCR standard curve. **(D)** *gltA*-duplex ddPCR standard curve (the target gene concentration corresponding to the amplification curve is 3 from left to right were 3 × 10^−1^, 3 × 10^−2^, 3 × 10^−3^, and 3 × 10^−4^ ng/μL).

The standard curves, as shown in Figures 4A,B, plotted the logarithmic values of the bacterial fluid concentrations measured against the Ct values obtained from amplification. The dual qPCR had a good linear relationship within the concentration range of 3 × 10^2^–3 × 10^−3^ ng/μL, and the coefficients of determination (*R*^2^) were 0.9772 and 0.9784, respectively. However, no amplification occurred with the bacterial fluid concentration at 3 × 10^−4^ ng/μL. Hence, the LOD of the dual qPCR established in this study is 3 × 10^−3^ ng/μL.

A standard curve was drawn using the logarithmic values of the ideal copy number and the actual copy number as the coordinates. The results of the dual ddPCR detection of OXA-23 and *gltA* positive sample concentration gradients showed that within the concentration range of 3 × 10^−1^–3 × 10^−4^ ng/μL, the number of positive droplets in the positive samples decreased with the target gene concentration and showed a good regularity (Figures 4C,D). The coefficients of determination (*R*^2^) were 0.9976 and 0.9949, respectively. Moreover, 100% detection was achieved at each concentration level. Therefore, the LOD of dual ddPCR for OXA-23 and *gltA* is 3 × 10^−4^ ng/μL, with measured copy numbers of 0.08 and 0.17 copies/μL, respectively.

When verifying the upper limit of the linear range in ddPCR, reaction systems with DNA concentrations of 300 and 30 ng/μL only had positive droplets. The template concentration was too high (>1,000,000) and was affected by the Poisson distribution, so its droplet count could not be accurately quantified, thus not included in the linear range.

### 3.5 Accuracy test

Three different concentrations of positive bacterial DNA were used for accuracy test of the dual ddPCR reaction system. As shown in Table 2, the CV values measured from the two gene concentrations of 3.0 × 10^−4^ ng/μL were less than 25%. Therefore, the dual ddPCR detection method for OXA-23 and *gltA* demonstrates good reproducibility with very low sample concentration.

**Table 2:** Precision of the dual ddPCR assay.

Sample		Average (copies/μL)	Standard deviation	CV (%)
3 × 10^−1^ ng/μL	OXA-23	1480.33	26.54	1.79
	gltA	718.33	24.23	3.37
3 × 10^−2^ ng/μL	OXA-23	6.2	0.45	7.33
	gltA	3.57	0.7	19.74
3 × 10^−3^ ng/μL	OXA-23	0.77	0.04	4.88
	gltA	0.72	0.14	19.55
3 × 10^−4^ ng/μL	OXA-23	0.27	0.06	23.20
	gltA	0.32	0.07	23.46

### 3.6 Results of clinical specimen verification

All 37 clinical samples were analyzed by ddPCR, blood culture, and drug sensitivity test. As shown in Table 3, seven CSAB samples tested positive only for *gltA*, 28 CRAB tested positive for both *gltA* and OXA23, the negative control, and the *Escherichia coli* tested positive for neither *gltA* nor OXA23. The ddPCR results were consistent with the clinical blood culture identification and antibiotic sensitivity test. Therefore, the duplex ddPCR method developed in this study can specifically detect AB and simultaneously determine the carbapenem resistance in BSIs. The quantitative results were presented in Supplementary Table 6.

**Table 3:** Clinical validation of the dual ddPCR assay.

ddPCR	Number of bacterial	OXA-23 (copies/μL)	gltA (copies/μL)	Drug sensitivity results (phenotype)
BC				
Negative	1	−	−	−
*Escherichia coli*	1	−	−	Carbapenem sensitive
CSAB	7	−	+	Carbapenem sensitive
CRAB	28	+	+	Carbapenem resistance

## 4 Discussion

Blood culture is the gold standard for detecting pathogens in BSIs; however, it has a lengthy turnaround time and relatively low sensitivity. Tabak et al. (2018) conducted a study of 165,593 blood samples from 13 hospitals in the United States, and found that the average time to identify BSIs pathogens using traditional blood culture was 44.0 h, with a sensitivity of approximately 70% in critically ill patients. To overcome the shortcomings of blood culture in BSIs diagnosis, we developed a culture-independent ddPCR method, which can accurately identify whether AB and its drug-resistant strains exist in BSI patient blood samples within 3–5 h. The ddPCR can directly detect and quantify CRAB rapidly. The ddPCR method requires 30 min to extract DNA from blood samples, approximately 3.5 h from droplet generation, PCR amplification to flow analysis, and 30 min for data analysis. The entire process does not exceed 5 h, which is far less than the 3–5 days required for domestic blood culture, greatly reducing the time of diagnostic report issuance.

This ddPCR method showcases a highlight: it can both quantify AB infection in human blood and reveal its main drug resistance characteristics simultaneously. Firstly, the carbapenem resistance rate of AB shows a rising trend year by year, and it is difficult to be treated. Secondly, the proportion of CRAB detected from blood samples is increasing yearly (Hu et al., 2016). Thirdly, many domestic and foreign studies show that carrying the OXA-23 gene is greatly related to carbapenem resistance in AB (Fu et al., 2010). Therefore, this study designed and screened primers and probes (Supplementary Table 5) to detect CRAB from the blood with high specificity. It does not cross-react with human DNA and other common BSI bacteria, fungi, viruses, and other pathogenic microorganisms, and can be used for clinical sample detection.

The ddPCR used in this study has higher sensitivity and good accuracy. The ddPCR and qPCR all existed a good linear relationship. But for a bacterial solution concentration of 3.0 × 10^−4^ ng/μL, qPCR did not amplify while ddPCR detected it, indicating that the detection sensitivity of ddPCR is higher than qPCR, consistent with the research results of Mavridis et al. (2022). Moreover, the LOD of ddPCR is lower than traditional qPCR, reaching 5–6 copies per microliter, so it can detect trace pathogen DNA in the blood, providing guidance for precise early clinical medication (Galimberti et al., 2022).

The pathogenic microorganisms in BSIs are difficult to detect in the early stages of infection not only due to their low concentration, but also for the complex components of blood which may interfere when detection. ddPCR is a new generation of quantitative detection technology that has emerged in recent years. Based on the traditional PCR principle and Poisson distribution, it has higher sensitivity and stronger anti-interference ability, which can detect the target gene from complex backgrounds with minimal interference. In recent years, ddPCR has also been widely used to detect a variety of pathogens in BSIs, including AB and *Klebsiella pneumoniae* (Zheng et al., 2021), *C. parapsilosis* and *S. aureus* (Hu et al., 2021), HIV (Roberds et al., 2022), *Escherichia coli* and *Pseudomonas aeruginosa* (Wu et al., 2022), and *Enterococcus faecalis* (Lin et al., 2023).

However, ddPCR has its limitations. Its high cost means that ddPCR technology is not yet widespread in clinical practice and remains primarily in the research phase. Still, some manufacturers have begun to introduce a series of ddPCR test kits for clinical use. As ddPCR testing technology and equipment become indigenized, the testing cost will be significantly reduced. Given its excellent quantitative detection principle and anti-interference ability, it is expected to be widely used in the detection of pathogenic microorganisms in BSIs.

## 5 Conclusion

The dual ddPCR reaction system constructed in this study exhibits highspecificity and accuracy. The linear range is OXA-23: 2165–0.08 copies/μL and *gltA*: 1113–0.17 copies/μL. The LOD of ddPCR is 3 × 10^−4^ ng/μL, which is higher than that of qPCR. When applied to detect venous blood from both positive and negative blood culture patients, the results were consistent with clinical blood culture identification and drug sensitivity tests. This system shortens the reporting time and provides technical support for the earlier stage diagnosis and precise treatment of BSIs.

## Data availability statement

The datasets presented in this study can be found in online repositories. The names of the repository/repositories and accession number(s) can be found in the article/Supplementary material.

## Ethics statement

Ethical approval was not required for the studies on humans in accordance with the local legislation and institutional requirements because only commercially available established cell lines were used.

## Author contributions

XK: Methodology, Writing – original draft. DZ: Data curation, Writing – original draft. YZ: Conceptualization, Writing – original draft. LH: Data curation, Writing – original draft. ZW: Project administration, Writing – original draft. ZL: Methodology, Project administration, Writing – original draft. CG: Data curation, Formal Analysis, Writing – original draft. LY: Conceptualization, Methodology, Supervision, Writing – original draft, Writing – review & editing. JL: Writing – review & editing.
