# Supplementary material for: Development and clinical validation of a dual ddPCR assay for detecting carbapenem-resistant Acinetobacter baumannii in bloodstream infections
Source: Front Microbiol. 2024 Mar 5;15:1338395. doi: 10.3389/fmicb.2024.1338395 (PMC11000175; doi:10.3389/fmicb.2024.1338395)
Supplement: Supplementary file 1 [file Data_Sheet_1.PDF]

Supplemental Table 1. Summary of Ct values of three sets of primer amplification curves for OXA-23

OXA-23 Ct	First group	Second group	Third group
H2O	NA	NA	NA
CSAB-1	NA	NA	NA
CSAB-2	NA	NA	NA
MDRAB-1	18.01	18.02	17.07
MDRAB 2	17.06	17.27	16.25
XDRAB-1	23.18	23.02	21.92
XDRAB- 2	20.37	NA	18.53

Note: NA. means no amplification

Supplemental Table 2. Summary of Ct values of three sets of primer amplification curves for gltA

gltA Ct	First group	Second group	Third group
H2O	NA	NA	NA
CSAB-1	19.88	19.84	18.11
CSAB-2	15.93	16.79	15.51
MDRAB-1	18.71	18.78	18.05
MDRAB 2	18.61	18.74	18.19
XDRAB-1	23.56	23.71	23.31
XDRAB- 2	NA	22.22	20.93

Note: NA. means no amplification

Supplemental Table 3 PCR primers and probes sequence after screening

Genes	Sequence (5’→3’)
OXA-23	F：GAC ACT AGG AGA AGC CAT GAA GCT
	R：GCA TGA GAT CAA GAC CGA TAC G
	P：TCC CAG TCT ATC AGG AAC TTG CGC G
gltA	F：GAA AAT GTT GCT GAG TTC ATG GAA
	R：GGT GAC CGA AGC CCA TAA GTT
	P：AGT TAA ACG CAA AGA AG

Supplemental Table 4 Primers and probes specificity verification results of OXA-23

microorganism	duplex qPCR	singleplex ddPCR	duplex ddPCR
CSAB	0/3 (0)	0/3 (0)	0/3 (0)
MDRAB	2/2 (100)	2/2 (100)	2/2 (100)
XDRAB	3/3 (100)	3/3 (100)	3/3 (100)
27 other strains	0/27 (0)	0/27 (0)	0/27 (0)

Supplemental Table 5 Primers and probes specificity verification results of gltA

microorganism	duplex qPCR	singleplex ddPCR	duplex ddPCR
CSAB	3/3 (100)	3/3 (100)	3/3 (100)
MDRAB	2/2 (100)	2/2 (100)	2/2 (100)
XDRAB	3/3 (100)	3/3 (100)	3/3 (100)
27 other strains	0/27 (0)	0/27 (0)	0/27 (0)

Supplemental Table 6 Specificr results of phenotype and genotype of the carbapenem resistance between BC and ddPCR

ddPCR / blood culture	OXA-23 （copies/μL）	gltA （copies/μL）	Drug sensitivity results Carbapenem resistance
negative	0	0	-
Escherichia coli	0	0	S
S1	0	93	S
S2	0	>1000000	S
S3	0. 6	>1000000	S
S4	0	>1000000	S
S5	0. 1	>1000000	S
S6	0	11000	S
S7	0. 1	10200	S
M1	54. 1	1130	R
M2	21. 3	19. 7	R
M3	9800	>1000000	R
M4	9100	>1000000	R
M5	9400	>1000000	R
M6	9300	>1000000	R
M7	6750	11000	R

M8	9800	4760	R
M9	5380	5590	R
M10	9700	11000	R
M11	6720	3030	R
M12	6750	11000	R
X1	99	21	R
X2	1169	578	R
X3	837	420	R
X4	>1000000	7310	R
X5	11000	>1000000	R
X6	3930	6360	R
X7	1687	496	R
X8	8900	9500	R
X9	>1000000	>1000000	R
X10	4140	2035	R
X11	>1000000	>1000000	R
X12	11200	7120	R
X13	>1000000	10300	R
X14	8200	4760	R
X15	>1000000	>1000000	R
X16	6750	8800	R

First column: S1-7: CSAB, M1-12: MDRAB, X1-16: XDRAB； Fourth column: S：Carbapenem sensitive，R：Carbapenem resistance
